# Premature Infants 750–1,250 g Birth Weight Supplemented with a Novel Human Milk-Derived Cream Are Discharged Sooner

**DOI:** 10.1089/bfm.2015.0166

**Published:** 2016-04-01

**Authors:** Amy B. Hair, Erynn M. Bergner, Martin L. Lee, Alvaro G. Moreira, Keli M. Hawthorne, David J. Rechtman, Steven A. Abrams, Cynthia L. Blanco

**Affiliations:** ^1^ Section of Neonatology, Department of Pediatrics, Baylor College of Medicine, Texas Children's Hospital, Houston, Texas.; ^2^ Prolacta Bioscience, Industry, California.; ^3^ Department of Pediatrics, University of Texas Health Science Center, San Antonio, Texas.; ^4^ Department of Pediatrics, Dell Medical School at the University of Texas at Austin, Austin, Texas.

## Abstract

***Objective:*** Infants may benefit from early nutritional intervention to decrease hospital stay. To evaluate the effects of adding a human milk (HM)-derived cream (Cream) product to a standard feeding regimen in preterm infants.

***Materials and Methods:*** In a prospective multicenter randomized study, infants with birth weights 750–1,250 g were assigned to a Control or Cream group. The Control group received a standard feeding regimen consisting of mother's own milk or donor HM with donor HM-derived fortifier. The Cream group received the standard feeding regimen along with an additional HM-derived cream supplement when the HM they received was <20 kcal/oz. Primary outcomes of this secondary analysis included comorbidities, length of stay (LOS), and postmenstrual age (PMA) at discharge.

***Results:*** We enrolled 75 infants (Control *n* = 37, Cream *n* = 38) with gestational age 27.7 ± 1.8 weeks and birth weight 973 ± 145 g (mean ± SD). After adjusting for gestational age, birth weight, and presence of bronchopulmonary dysplasia (BPD), the Cream group had a decreased PMA at discharge (39.9 ± 4.8 versus 38.2 ± 2.7 weeks, *p* = 0.03) and LOS (86 ± 39 versus 74 ± 22 days, *p* = 0.05). For 21 infants with BPD, these values trended toward significance for PMA at discharge (44.2 ± 6.1 versus 41.3 ± 2.7 weeks, *p* = 0.08) and LOS (121 ± 49 versus 104 ± 23 days, *p* = 0.08).

***Conclusions:*** Very preterm infants who received an HM-derived cream supplement were discharged earlier. Infants with BPD may have benefited the most.

## Introduction

Human milk (HM) is accepted as the ideal source of nutrition for all infants, including those born prematurely. Its use provides benefits related to host defense, gastrointestinal maturation, infection rate, neurodevelopmental outcomes, and long-term cardiovascular and metabolic disease.^1^ An exclusive HM diet significantly decreases the rates of necrotizing enterocolitis (NEC), sepsis, days of parenteral nutrition, and death.^2–4^ As such, the American Academy of Pediatrics^5^ recommends that mother's own milk or donor HM should be used as the foundation of enteral feeds for all very low birth weight (VLBW) infants.

However, due to the increased requirements of VLBW infants, HM cannot meet the nutritional needs of this population without fortification.^6,7^ Recent data have shown that the average energy content of a nationwide sample of donor HM was 19 kcal/oz with 25% of samples falling below 17.3 kcal/oz and 65% of the samples below 20 kcal/oz.^6^ Another similar analysis of both donor and mother's own milk demonstrated that most samples had nutrient contents below the recommended values for preterm milk composition with 79% having a fat content <4 g/dL, 56% having protein content <1.5 g/dL, and 67% having an energy density <67 kcal/dL.^8^

Optimal nutrition is especially important for VLBW infants with bronchopulmonary dysplasia (BPD). Despite an estimated 20–40% increase in caloric needs,^9,10^ infants with BPD receive more fluid and less energy than their healthy counterparts in the first weeks of life.^11,12^ Future nutrition and growth can then be further compromised by the need for fluid restriction, diuretics, and postnatal steroids to manage this disease.^10^

A novel HM-derived cream supplement (Cream) allows for the enhancement of the energy density of feeds without a considerable increase in the volume of feeds given to the infant. We hypothesized that extremely premature infants, especially those with higher nutritional needs, who received supplementation with Cream would have improved outcomes and decreased length of stay (LOS).

## Materials and Methods

In this multicenter trial, infants were fed an exclusive HM diet according to the investigative site's standard feeding protocol. This diet included mother's own milk or pasteurized donor HM fortified with pasteurized donor HM-derived fortifier, Prolact+H^2^MF (Prolacta Bioscience, Industry, CA). Details of this study and growth outcomes have been reported.^13^ After informed consent was obtained, infants were randomized in blocks of four into two groups. Investigators were blinded to block size. Once the infants began tolerating fortified enteral feeds (at ∼100 mL/kg/d), milk analysis with a near infrared milk analyzer (Spectrastar 2400RTW; Unity Scientific, Brookfield, CT) began. Infants randomized to the Cream group were supplemented with the 2.5 kcal/mL HM-derived cream supplement (Prolact CR) whenever their mother's own milk or donor HM was found to be below 20 kcal/oz. Cream was added through the procedure outlined previously,^13^ such that if the base HM was 19 kcal/oz, 2 mL of the HM-derived cream Supplement was added to 98 mL of mother's own milk or donor HM. This study was approved by the Institutional Review Board of Baylor College of Medicine and Affiliated Hospitals and The University of Texas Health Science Center (San Antonio, TX). It was registered with ClinicalTrials.gov (NCT01487928).

Neonatal demographics and hospital courses were obtained from the medical record. Outcome variables collected included medically (indomethacin or ibuprofen course) or surgically managed patent ductus arteriosus (PDA), blood culture-proven late-onset sepsis, NEC (defined as Stage 2 NEC or greater by the modified Bell Criteria^14^), BPD (defined as oxygen therapy/positive pressure ventilation at 36 weeks postmenstrual age (PMA) to maintain an adequate range of oxygen saturation^15^), mortality, LOS, PMA at discharge, and growth parameters (weight, length, and head circumference). All growth parameters were plotted on the Olsen curve^16^ to obtain growth percentiles. Discharge criteria were defined by the individual attending physicians at the investigative sites. As this was a secondary analysis of a growth study, discharge criteria were not standardized in the original protocol. However, weight was not a specific criterion for discharge. As infants with BPD have increased nutritional needs,^9,10^ we performed a subgroup analysis to compare the control infants with BPD to the intervention group with BPD. The analyses in this article were performed in addition to those previously published with this study.^13^

We performed a univariate statistical analysis using the Wilcoxon rank-sum test for quantitative data. For the categorical outcomes in the Cream versus Control comparison, the chi-square test for homogeneity, Fisher's exact test, or its multinomial equivalent was employed. The analyses for LOS and PMA at discharge utilized a multivariate linear model that controlled for gestational age, birth weight, and presence of BPD along with the interaction of BPD and cream use, as the main effects of study group and BPD could have had a nonlinear component represented by the multiplicative interaction of the two. All statistical calculations were performed utilizing an intent-to-treat analysis. Statistical significance was defined as *p* < 0.05.

The study was not powered to detect effects on comorbidities, LOS, or PMA at discharge. As has been noted, this trial was originally focused and powered on growth outcomes.

## Results

A total of 78 infants of 750 to 1,250 g were randomized; 3 of these infants were excluded from analysis (1 due to sepsis and a subsequent bowel obstruction before the start of milk analysis, 1 due to clinically significant congenital heart disease and chromosomal abnormality, and 1 due to intestinal perforation before the start of fortified feeds) as their underlying conditions placed undue influence on the primary outcomes of this study. Thus, 75 infants (Control *n* = 37, Cream *n* = 38) were evaluated. There were no significant differences in the characteristics of the Control and Cream intervention groups at study enrollment with the exception of race (Table 1). Twenty-one infants with BPD were also evaluated in a subgroup analysis. The BPD subgroup did not exhibit any significant difference in the study groups with respect to baseline characteristics (Table 2).

**Table 1.** Infant Demographics and Characteristics (Mean ± SD)

	*Control group (*n* = 37)*	*Cream group (*n* = 38)*	p
Birth weight, g	973 ± 152	973 ± 140	0.996
SGA at birth, %	18.9	13.2	0.504
Gestational age, weeks	27.7 ± 2.0	27.7 ± 1.6	0.93
Gender, % male	56.8	47.4	0.42
Race, % Hispanic/White/Black/Other	51.4/21.6/21.6/5.4	21.0/23.7/47.4/7.9	0.03
APGAR at 5 minutes	7 ± 2	7 ± 2	0.39
Mechanical ventilation, %	18.9	15.8	0.72
Antenatal steroids, %	81.1	79.0	0.82

Analyses of birth weight and gestational age used the two sample *t*-test; all others were based on the chi-square test for homogeneity.

SD, standard deviation; SGA, small for gestational age.

**Table 2.** BPD Subgroup Demographics

	*Control group with BPD (*n = 12)	*Cream group with BPD (*n = 9)	p
Birth weight, g	949 ± 145	855 ± 104	0.12
SGA at birth, %	8.3	0.0	1.0
Gestational age, weeks	27.0 ± 1.7	26.7 ± 1.4	0.60
Gender, % male	66.7	44.4	0.40
Race, % Hispanic/White/Black/Other	58.3/8.3/25.0/8.3	11.1/22.2/44.4/22.2	0.16
APGAR at 5 minutes	7 ± 2	7 ± 3	0.40
Mechanical ventilation, %	41.7	33.3	1.0
Antenatal steroids, %	91.7	66.7	0.27

All analyses of categorical data in this table used Fisher's exact test or its multinomial equivalent; analyses of birth weight and gestational age used the Wilcoxon rank-sum test.

BPD, bronchopulmonary dysplasia.

The clinical outcomes are listed in Table 3. These outcomes were notable for a significantly shorter PMA at discharge in the Cream group (38.2 ± 2.7 weeks) compared to the Control group (39.9 ± 4.8 weeks), with a *p*-value of 0.03 after employing the linear adjustment model described above. LOS for infants that received cream was also shorter than those who did not receive cream (74 ± 22 days for the Cream group and 86 ± 39 days for the Control Group, *p* = 0.05, using the adjustment model). Data for the infants with BPD trended toward a shorter LOS and earlier PMA at discharge (Table 4). In this subset, infants in the Cream group were discharged from the hospital an average of 17 days sooner (LOS 104 ± 23 days for the BPD Cream group and 121 ± 49 days for the BPD Control group, *p* = 0.08). The PMA at discharge of the BPD Cream group was an average of 2.9 weeks earlier (PMA at discharge 41.3 ± 2.7 weeks for the BPD Cream group and 44.2 ± 6.1 weeks for the BPD Control group, *p* = 0.08).

**Table 3.** Clinical Outcomes of Study Infants

	*Control group (*n = 37)	*Cream group (*n = 38)	p
PDA ligation, %	8.1	2.6	0.36
PDA treated medically, %	27.0	29.0	0.85
Sepsis, %	5.4	7.9	1.0
Necrotizing enterocolitis, %	0	0	—
Bronchopulmonary dysplasia, %	32.4	23.7	0.40
Death, %	0	0	—
Length of stay, days	86 ± 39	74 ± 22	0.05^a,b^
PMA at discharge, weeks	39.9 ± 4.8	38.2 ± 2.7	0.03^a,c^

^a^ General linear model with all of the data and including main effects of group (Cream versus Control), BPD, the interaction between the two, and gestational age and birth weight as covariates in an adjustment model.

^b^ *p* = 0.17 by two-sample *t*-test on log-transformed values.

^c^ *p* = 0.07 by two-sample *t*-test. All others by chi-square test for homogeneity.

PDA, patent ductus arteriosus; PMA, postmenstrual age.

**Table 4.** Clinical Outcomes of Infants with BPD

	*Control group with BPD (*n* = 12)*	*Cream group with BPD (*n* = 9)*	p
PDA ligation, %	16.7	11.1	1.0
PDA treated medically, %	41.7	66.7	0.29
Sepsis, %	16.7	11.1	1.0
Necrotizing enterocolitis, %	0	0	—
Death, %	0	0	—
Length of stay, days	121 ± 49	104 ± 23	0.08^a,b^
PMA at discharge, weeks	44.2 ± 6.1	41.3 ± 2.7	0.08^a,c^

Fisher's exact test for categorical variables; Wilcoxon rank-sum test for quantitative variables (except for linear adjustment model).

^a^ General linear model with all of the data and including main effects of group (Cream versus Control), BPD, the interaction between the two, and gestational age and birth weight as covariates in an adjustment model.

^b^ *p* = 0.32 by two-sample *t*-test on log-transformed values.

^c^ *p* = 0.14 by two-sample *t*-test.

No significant difference was noted in the rates of BPD, late-onset sepsis, or PDA requiring intervention between the two groups. The percentage of small for gestational age infants (<10th percentile on the Olsen Curve^16^) at 36 weeks PMA did not significantly differ between the groups. There were no recorded deaths or episodes of NEC in this study.

## Discussion

We found that preterm infants who received the novel HM-derived cream supplement had a significantly earlier PMA at discharge and trend toward decreased LOS when compared to those who did not receive the cream supplement. These benefits also trended toward significance in the BPD subpopulation.

Recent analyses show fat to be the most variable component in HM, accounting for decreases in energy density.^6,7^ Thus, this study design compensates for this lower lipid content in the subject's base milk and enhances the exclusive HM diet with adequate fortification that is recommended for premature infants.^1–3^

Targeted individual fortification of HM has also emerged as an approach to provide infants with sufficient nutritional support for growth and development, while allowing them to receive the immune benefits of HM. Using a medium-chain triglyceride oil and a bovine-derived HM fortifier, de Halleux and Rigo^8^ observed that this method allowed for both maintenance of energy balance and adherence to the recommended values of individual nutrients in HM. The cream supplement may be useful in further investigations into this approach as its derivation from HM allows for the addition of HM lipids to the infant's diet. The advantage of utilizing an HM-derived lipid supplement over medium-chain triglyceride oils is the potential for improved tolerance, while avoiding exposures to allergens from the source of the current available oils (i.e., soy, sunflower).

With these nutritional benefits, cost containment also exists as one of the most apparent advantages to an earlier discharge. By analyzing the 2001 Nationwide Inpatient Sample from the Healthcare Cost and Utilization Project, Russell et al.^17^ found that, while a diagnosis of prematurity or low birth weight represented only 8% of infant hospitalizations, these diagnoses accounted for 47% of the costs (∼$5.8 billion). Furthermore, illness-related costs associated with BPD are the highest of the common comorbidities of prematurity, reaching 2.3 times the amount required to care for a gestational age-matched infant without BPD.^18^ We did not include a cost analysis in this study; however, the cost of the cream supplement is relatively inexpensive, and we infer cost savings with decreasing LOS.

The trend toward a decrease in LOS noted for the infants with BPD may be attributed to the vital role that adequate nutrition plays in weight gain and presumed lung growth and development.^19,20^ Human and animal models have shown that caloric restriction significantly reduces alveolar number and surface area in addition to collagen deposition in the lungs.^21–24^ Furthermore, HM lipids promote synthesis and secretion of pulmonary surfactant^25^ and improve the bioavailability of fat-soluble vitamins,^9^ including Vitamin A, which have independently been shown to reduce the incidence of BPD.^12,25,26^ Delivering additional lipids to meet the increased caloric needs of infants with BPD may also be advantageous as the metabolism of fat produces less carbon dioxide compared with carbohydrates.^9,10^ As a result, the decreased caloric intake by the Control subjects in our study may have interfered with their ability to continue lung development in the postnatal period, resulting in possible diminished pulmonary function.^9^

This cream formulation, at 2.5 kcal/mL, also allows for a substantial amount of calories to be added without a considerable increase in total feeding volume. Fluid restriction is especially important in the management of VLBW infants due to their predisposition to developing pulmonary edema.^9^ It has been postulated that higher fluid intake inhibits the process of extracellular fluid contraction after birth resulting in decreased lung compliance and need for more ventilator support that may damage the lung tissue and cause disease.^27^ As such, greater fluid intake and less weight loss in the first 10 days of life have been demonstrated to increase an infant's risk of developing BPD.^20,27^ Thus, although no significant difference in the rate of BPD was detected in this analysis, larger studies that investigate mechanisms to provide safe calorie-dense feeds to VLBW infants and the subsequent pulmonary outcomes of these infants may be warranted. Supplements such as the one studied here may be useful in such investigations.

This study was limited by the small sample size and, as a secondary analysis, was not powered to measure the primary outcomes of this investigation. As such, further studies would be needed to detect any difference in the rate of comorbidities or clinical outcomes of the BPD subgroup. In addition, the donor HM used by one investigative site was specifically formulated to provide a minimum of 20 kcal/oz. As a result, 13% (5/38) of infants randomized to the Cream group did not receive the intervention at any point during the trial. Those infants receiving mainly donor HM for their base milk supply may have falsely elevated the average energy density of the base milk of both the study and intervention group, minimizing the difference between them. In addition, the near-infrared milk analyzer for milk analysis used a secondary method for the measurement of HM and after comparison with primary AOAC^©^ methods, despite calibration to bias samples, we found that there was a 1.2 kcal/oz overestimation of the caloric content of the HM samples.^13^ Therefore, more samples of milk in the intervention group likely qualified for cream than actually received it potentially, minimizing the clinical effect. Also of note, one of the two investigative sites had unblinded randomization due to logistical reasons. We do not feel that this is clinically relevant, but it is a limitation of the study.

## Conclusion

We found a significantly earlier PMA at discharge and trend toward shorter LOS of 750–1250 g infants receiving the cream supplement. Although this difference only approached significance for the infants with BPD, this subset of infants seemed to be impacted. These findings have large implications in decreasing healthcare costs, improving individual fortification strategies, and enhancing overall nutrition of premature infants. Larger studies utilizing this supplement within an exclusive HM diet are needed to assess the full impact of its clinical effect, especially in relation to the outcome of infants with comorbidities with specific nutritional challenges such as BPD.
